# Molecular Insights into the Nociceptive Modulation by Palmitoylethanolamide and *Equisetum arvense* Extract: An In Vitro Study Across the Blood–Brain Barrier

**DOI:** 10.3390/nu17121998

**Published:** 2025-06-13

**Authors:** Simone Mulè, Rebecca Galla, Sara Ferrari, Marco Invernizzi, Francesca Uberti

**Affiliations:** 1 Department for Sustainable Development and Ecological Transition, University of Piemonte Orientale (UPO), 13100 Vercelli, Italy; simone.mule@uniupo.it (S.M.); sara.ferrari@uniupo.it (S.F.); 2 Noivita Srls, Spin Off, University of Piemonte Orientale, Strada Privata Curti 7, 28100 Novara, Italy; rebecca.galla@uniupo.it; 3 Physical and Rehabilitation Medicine, Department of Health Sciences, University of Eastern Piedmont, 28100 Novara, Italy; marco.invernizzi@med.uniupo.it; 4 Translational Medicine, Dipartimento Attività Integrate Ricerca e Innovazione (DAIRI), Azienda Ospedaliero-Universitaria (AOU) SS, Antonio e Biagio e Cesare Arrigo, 15121 Alessandria, Italy

**Keywords:** transwell^®^ system, 3D in vitro model, neuroinflammation, nociception, endocannabinoids pathway, nutraceutical approach, plant extract

## Abstract

**Background:** The blood–brain barrier (BBB) plays a critical role in protecting the central nervous system (CNS) but also limits drug delivery. Insufficient knowledge of how the CNS promotes the onset and maintenance of peripheral neuropathic pain limits therapeutic methods for the treatment of persistent neuropathic pain. Thus, this study aimed to evaluate the ability of a novel combination of Palmitoylethanolamide (PEA) and *Equisetum arvense* L. (*Equisetum A.L.*) to cross the BBB and modulate nociceptive pathways. **Methods:** Using a humanised in vitro BBB tri-culture model, the permeability, cytotoxicity, and integrity of the barrier were assessed after exposure to two different PEA forms, PEA ultramicronized (PEA-um) and PEA80mesh, *Equisetum A.L.*, and a combination of the last two samples. The samples exhibited no cytotoxicity, maintained tight junction integrity, and efficiently crossed the blood–brain barrier (BBB), with the combination displaying the highest permeability. The eluate from the BBB model was then used to stimulate the co-culture of CCF-STTG1 astrocytes and SH-SY5Y neurons pre-treated with H_2_O_2_ 200 µM. **Results:** Treatment with the combination significantly increased cell viability (1.8-fold, *p* < 0.05), reduced oxidative stress (2.5-fold, *p* < 0.05), and decreased pro-inflammatory cytokines (TNFα, IL-1β) compared to single agents. Mechanistic analysis revealed modulation of key targets involved in pain pathways, including decreased FAAH and NAAA activity, increased levels of endocannabinoids (AEA and 2-AG), upregulation of CB2 receptor expression, enhanced PPARα activity, and reduced phosphorylation of PKA and TRPV1. **Conclusions:** These findings suggest that the combination of PEA and *Equisetum A.L.* effectively crosses the BBB and exerts combined anti-inflammatory and analgesic effects at the CNS level, suggesting a possible role in modulating neuroinflammatory and nociception responses.

## 1. Introduction

The blood–brain barrier (BBB) is a key microvascular structure in the central nervous system (CNS) that regulates the selective transport of molecules and cells between the blood and brain tissue [1,2,3,4]. This restricted barrier enables BBB endothelial cells to regulate CNS homeostasis, which is essential for neuronal function and protection against toxins, infections, inflammation, damage, and disease [2,5]. Furthermore, a less favourable response to treatment interventions and a more severe cognitive decline are associated with higher BBB permeability [6]. In vitro BBB models are essential for studying barrier properties, with the co-culture model of endothelial cells (ECs) in association with cultured pericytes and astrocytes being one of the simplest and most feasible approaches. This model provides a paracellular seal and elucidates the functions of the epithelial cell surface (ECS), allowing for the accurate examination of the permeability rate of substances like sodium fluorescein [7,8,9,10]. Significant alterations in BBB permeability have been observed between healthy animals and those exposed to hypoxia/reoxygenation (*H*/*R*) or peripheral inflammatory pain (PIP). These disorders, characterised by pain, inflammation, and hyperalgesia, are crucial components of many CNS diseases and can significantly impact how analgesics targeting nociception are delivered to the brain [11,12,13]. In recent years, scientific research has focused on understanding how the CNS promotes the onset and maintenance of peripheral neuropathic pain, aiming to improve therapeutic methods for treating persistent neuropathic pain by targeting nociception and pain [14]. The brain mechanism that detects and processes unpleasant stimuli is called nociception; it takes place without conscious awareness and causes target organs to react autonomically [15]. Nociceptive signalling occurs at the molecular level when specific molecules at nerve terminals detect painful stimuli and initiate sensory transduction, which converts damaging energy into ionic currents. This is followed by conduction, which occurs when action potentials propagate along afferent fibres and cause neurotransmitter release in the dorsal horn, relaying the signal to the brain [16]. At the level of the CNS, the information that constitutes nociception once encoded is converted into pain which, unlike nociception, is the subjective experience of that encoded information [17]. Crucial in this scenario are channel 1 of the transient vanilloid receptor potential (TRPV1) and the cannabinoid receptor 2 (CB2R) at the central level, as well as acidamide hydrolase (FAAH), both of which act on arachidonic-hydrolase type molecules, which are responsible for pain awareness. Indeed, studies have shown that the inhibition of FAAH and TRPV1 can produce anti-nociception in in vivo models, as demonstrated in vitro [18,19,20]. At the same time, the increased expression of CB2R at the neuronal level has also been outlined as crucial for modulating the nociceptive mechanism and endocannabinoid pathway [21].

Natural substances, such as nutraceuticals, have long been utilised in traditional medicine for this purpose, and there is a wealth of data to support their ability to reduce oxidative stress and chronic inflammation associated with nociception and pain [22]. A natural product of the food industry, nutraceuticals may have physiological advantages when consumed. They are utilised to postpone ageing, prevent chronic illnesses, and enhance health [23]. Palmitoylethanolamide (PEA) exhibits anti-inflammatory, immunomodulatory, and neuroprotective properties, which are mediated by various molecular targets throughout the central and peripheral nervous systems (PNS), according to current data. It penetrates the BBB, primarily accumulating in the hypothalamus, pituitary, brain stem, cerebellum, and brain cortex [6]. PEA mitigates the increased permeability of the blood–brain barrier observed in an in vitro model simulating ischemic injury through oxygen–glucose deprivation and subsequent reperfusion (OGD/R) [24]. Neurons and glial cells synthesise PEA in response to consumer demand in the CNS. It is implicated in endogenous neuroprotective pathways activated in response to tissue damage or inflammation [25], suggesting that its exogenous administration may enhance inflammation resolution and restoration of tissue homeostasis. More recently, PEA has been shown to elicit microglial alterations associated with enhanced migration and phagocytic activity [26]. In human tissues, PEA levels are mainly determined by the enzyme-mediated production of *N*-palmitoylethanolamine-phospholipids and their ensuing degradation by either FAAH [27] or, in the context of inflammation, *N*-acylethanolamine-hydrolysing acid amidase (NAAA) [28]. Considering NAAA has been shown to hydrolyse PEA more so than other acylethanolamides (AEs), selective inhibitors of NAAA are predicted to have anti-inflammatory and analgesic properties and raise local levels of endogenous PEA. The impacts produced by centrally elevated AE levels are now better understood because of the availability of specific FAAH inhibitors [29]. All AEs, including the traditional cannabinomimetic arachidonoylethanolamide or anandamide (AEA) and 2-arachidonoylglycerol (2-AG), can increase their levels by these inhibitors. Consequently, their simultaneous increase could contribute to several pharmacological effects, such as analgesic response [25]. PEA has anticonvulsant, antipyretic, antiepileptic, immunomodulatory, neuroprotective, analgesic properties. Its main mechanism of analgesia and anti-inflammatory effects is the activation of peroxisome proliferator-activated receptor alpha (PPARα), which reduces inflammation and pain by inhibiting pro-inflammatory cytokines, such as tumour necrosis factor α (TNF-α), IL-6, and interleukin-1β (IL-1β). PEA also inhibits mast cell degranulation and modulates microglial activation. Furthermore, while PEA has a low affinity for the cannabinoid receptors 1 (CB1R) and CB2R, and hence cannot be classified as a conventional endocannabinoid, it is an endogenous endocannabinoid-like molecule that targets cannabinoid pathways [30]. Comparably, endocannabinoids also target TRPV1, which PEA can indirectly stimulate. Variations in the phosphorylation state of TRPV1 are necessary for its activity and are mediated by regulatory proteins, including ATP, protein kinase A (PKA), and protein kinase C (PKC). It appears that phosphorylation is necessary for TRPV1 activation/sensitisation, contributing to the transmission of pain, inflammation, and neurotoxicity. On the other hand, blocking this pathway results in desensitisation, which supports the analgesic and anti-inflammatory effects of TRPV1 agonists [31].

One of the limiting factors of PEA is its low oral absorption rate in aqueous vehicles, which results in low oral bioavailability. Further exogenous supplementation is required in pathological circumstances, as PEA, which can be found in various food sources, is insufficient for optimal intake [32]. Indeed, the native PEA’s large particle size (~2000 μm) limits absorption and bioavailability, reducing its effectiveness. Micronized (mPEA) and ultramicronized (umPEA) forms improve cellular uptake and counteract neuroinflammation [33,34]. These limitations can easily be addressed through advances in technology such as micronization or ultramicronization or by combining PEA with particular solvents [35,36,37,38]. At the same time, combining mPEA and umPEA with natural compounds like polydatin (e.g., mPEAPol) can enhance their biological activity, such as anti-inflammatory, antioxidant, and protective actions in a tissue-specific manner [34]. Indeed, PEA does not have the direct antioxidant ability to counteract the production of free radicals, which can harm proteins, lipids, and DNA. Studies have focused on combining PEA with natural molecules that can counteract oxidative stress and peroxidation processes. Key examples include flavonoids like luteolin, polidatin, quercetin, and silymarin, which exhibit diverse pharmacological properties and therapeutic potential [39,40,41].

Some of these active ingredients have been identified in some botanical extracts, including *Equisetum arvense* L. (*Equisetum A.L.*), which has been associated with pharmacological properties [42]. *Equisetum A.L.* represents a valuable example as an antioxidant. Its phytochemical constituents include flavonoids, phenolic acids, alkaloids, phytosterols, tannins, and triterpenoids promoting a neuroprotective action [43,44]. The extract of *Equisetum A.L.* also showed an anti-nociceptive effect given that the sterol fraction of the extract contains β-sitosterol, campesterol, and isofucosterol [45], which are all compounds with well-known anti-inflammatory effects. *Equisetum A.L.* extract has shown to significantly suppress the expression of COX-2 mRNA and inflammatory cytokines, such as TNF-α and IL-6, thus alleviating pain in a stomatitis model [46]. Polyphenolic derivatives from *Equisetum* species may offer further protection by activating the antioxidant transcription in the promoter regions of oxidative stress-inducing genes. These compounds are crucial for oxidative stress response and SIRT1 activation, mediating chronic pain from peripheral nerve damage. They can also influence cell signalling by reducing inflammation and inducing cell death [47]. In addition, some bioactive components present in *Equisetum A.L.* [48] have shown significant analgesic effects by modulating the activity of FAAH, such as quercetin and kaempferol. Various studies have shown that kaempferol suppresses the activity of FAAH [49,50]. At the same time, quercetin is reported to be a weak FAAH and lipoxygenases inhibitor, allowing the reduction of the degradation of AEA and 2-AG [51].

According to the details provided, the combination of PEA and *Equisetum A.L*. produced promising results. Moreover, the combination was shown not only to penetrate the intestinal barrier but also to reach the target region, modifying the process of nerve repair following Schwann cell damage and delivering an initial pain relief response [52]. Considering these outstanding results on the PNS, and based on the scientific evidence previously discussed, this study aims to evaluate the ability of the combination of PEA and *Equisetum A.L.* to cross the BBB and act on co-culture astrocytes/SH-SY5Y to induce beneficial effects. Indeed, this study aims to explore the analgesic effects promoted by PEA combined with *Equisetum A.L.* on directly related biological mechanisms at the CNS level associated with initiating the pain transmission pathway.

## 2. Materials and Methods

### 2.1. Preparation of Agents

**Figure A1:**
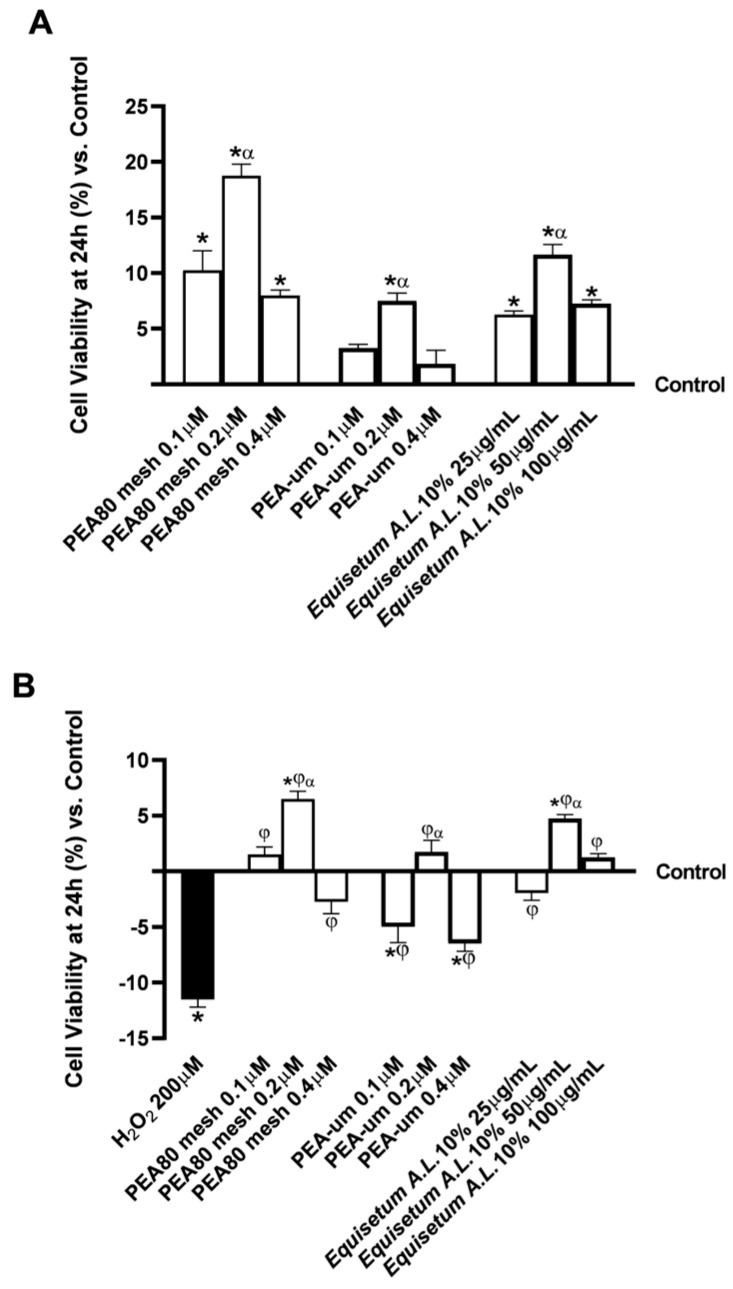
Dose–response study of individual test samples in terms of improvement and increase in cell viability (MTT test) after 24 h of treatment. (**A**), results after treatment with single agents at the level of the in vitro model of BBB; (**B**), results after treatment with single agents at the level of the co-culture model CCF-STTG1/SH-SY5Y. Data are expressed as means ± SD (%) of increased values derived from five independent experiments performed in triplicate, normalised to control values (0% line). For (**A**), * *p* < 0.05 vs. control; α *p* < 0.05 vs. other concentrations. For (**B**), * *p* < 0.05 vs. control; φ *p* < 0.05 vs. H_2_O_2_; α *p* < 0.05 vs. other concentrations.

The 0.2 µM 80mesh PEA (donated by Vivatis Pharma Italia srl, Gallarate, Italy) and 50 µg/mL *Equisetum A.L.* (titrated to 10% silica, donated by Vivatis Pharma Italia srl) alone and combined (named EquiPEA, Patent Nos. 102022000008066 and 102022000016404 by AGAVE Group, Bologna, Italy) were used to test their efficacy in crossing the BBB and reaching the CNS compared to the 0.2 µM ultramicronized PEA (PEA-um; donated by Vivatis Pharma Italia srl). PEA and *Equisetum A.L*. concentrations were obtained from the literature [53,54,55,56], confirmed by a previous study [52] and dose response study reported in Appendix A (Figure A1). All compounds under investigation were solubilised directly in Dulbecco’s modified eagle’s medium (DMEM; Merck Life Science, Rome, Italy) lacking phenol red, and supplemented with 0.5% foetal bovine serum (FBS), 2 mM L-glutamine, and 1% penicillin–streptomycin (*P*/*S*) (all from Merck Life Science, Rome, Italy). Similarly, hydrogen peroxide (H_2_O_2_) was added to the same supplemented medium used for the other treatments at a final concentration of 200 µM [4].

### 2.2. Cell Cultures

The effects of PEA-um, PEA80mesh, *Equisetum A.L.*, and their combination were tested on the CCF-STTG1 human astrocyte cell line (ATCC, Manassas, VA, USA), obtained from a 68-year-old astrocytoma patient. The cells were grown in RPMI medium (Merck Life Science, Rome, Italy) supplemented with 10% FBS, 2 mM HEPES, 2 mM L-glutamine, and 1% penicillin–streptomycin. Analyses included cytotoxicity, oxidative stress, and pathways related to inflammation and analgesia (FAAH, PPARα, CB2R, pTRPV1, pPKA, NAAA, AEA, and 2-AG). CCF-STTG1 cells were co-cultured with SH-SY5Y cells following a published protocol (see Section 2.9) [57]. Following published techniques, astrocytes were co-cultured with human umbilical vein endothelial cells (HUVECs) and primary human brain vascular pericytes (HBVPs) to simulate the BBB [58].

SH-SY5Y cells (ATCC, Manassas, VA, USA) were maintained in a 1:1 mixture of advanced DMEM F12 and advanced DMEM (GIBCO^®^, Thermo Fisher Scientific, Waltham, MA, USA), supplemented with 10% FBS. Cultures were incubated at 37 °C in a humidified atmosphere containing 5% CO_2_ [59]. Experiments were conducted using cells at passages from 3 to 20 [60,61].

Primary human cerebral vascular pericytes (HBVPs) cells from ScienCell Research Laboratories (Carlsbad, CA, USA) were grown in a pericyte medium with growth a supplement and 2% FBS (ScienCell™ Research Laboratories, Carlsbad, CA, USA) [25,58].

The HUVEC line, sourced from ATCC (Manassas, VA, USA), was cultured in flasks coated with 0.1% gelatin, utilising the endothelial growth medium-2 (EGM-2) enriched with 2% FBS, hydrocortisone (0.04%), human fibroblast growth factor beta (0.4%), vascular endothelial growth factor (0.1%), recombinant human insulin-like growth factor-I analogue (0.1%), ascorbic acid (0.1%), human epidermal growth factor (0.1%), Gentamicin sulphate–Amphotericin (0.1%), and heparin (0.1%) (all reagents from Lonza, Walkersville, MD, USA) [4]. Cells were employed between passages 3 and 6 at 37 °C in a 95% humidified air and 5% CO_2_ environment [62].

### 2.3. Experimental Protocol

**Figure 1:**
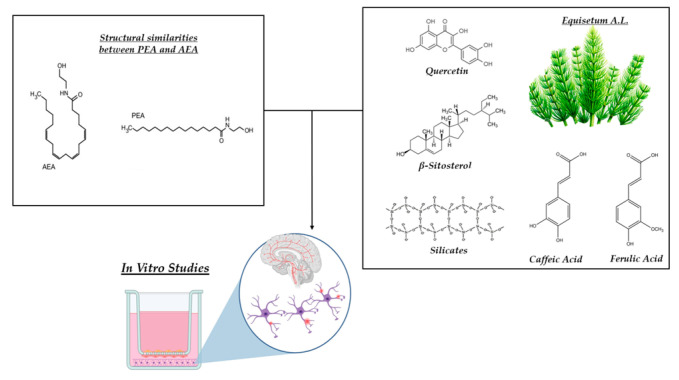
Illustration of the chemical structures of PEA and relevant bioactive components contained in *Equisetum A.L*. used during in vitro studies.

The experimental protocol was structured into 2 phases, aimed at evaluating the effects of the in vitro samples, not only on the BBB tri-culture model but also on the molecular mechanisms related to nociception. Figure 1 illustrates the chemical structural characteristics of PEA and the primary bioactive components of *Equisetum A.L.* that may impact the mechanisms under study. In the first phase of the experiment, the selected concentrations of PEA and *Equisetum A.L.*, which have been shown in a previous study to cross the intestinal barrier without inducing any damage or cytotoxic effects, thereby promoting exceptional effects of reducing inflammation and mechanisms connected to peripheral neuropathic pain [52], were used to test the effects on the BBB in vitro. An MTT assay (3-(4,5-dimethylthiazol-2-yl)-2,5-diphenyltetrazolium bromide) was used to measure cell viability, whereas cytochrome C-based ROS generation was employed to rule out harmful effects. In addition, the integrity status of the BBB was analysed by utilising a specific ELISA kit to detect the main TJ, such as claudin-5 and marveld. At the same time, the permeability test was performed in a time-course study, using a fluorescent probe to indicate the correct crossing of the samples through the BBB model.

In the second phase, at the end of the stimulation period, the eluate medium from the BBB tri-culture model was collected and used to stimulate the co-culture CCF-STTG1/SH-SY5Y for 24 h. This co-culture was pre-treated with H_2_O_2_ 200 µm for 30 min to mimic the inflammatory and damage conditions [63,64]. Later, as well as examining the modulations of viability (MTT assay) and oxidative stress (Cytochrome C) in terms of ROS production, at the level of the co-culture CCF-STTG1/SH-SY5Y’s lysates and supernatants, specific ELISA kits were used to characterise the following: inflammatory response (TNFα and IL-1β); the intracellular pathways directly related to the analgesic activity by analysing FAAH, NAAA, PPARα, CB2R, pTRPV1, pPKA, and the levels of AEA and 2-AG; the presence of the CB2 receptor by Western blot; and NAAA activity by competitive assay. These analyses were also conducted to understand how PEA-um, PEA80mesh, *Equisetum A.L.*, and PEA80mesh combined with *Equisetum A.L*. modulate intracellular PEA metabolism.

### 2.4. Blood–Brain Barrier (BBB) In Vitro Model

According to the protocol described in the literature, the astrocyte cell line was cocultured with HBVPS and HUVEC lines [58]. To establish a tri-culture Transwell^®^ model of the BBB, 12-well Transwell^®^ inserts were used. Type I collagen (150 μg/mL; Merck, Darmstadt, Germany) and poly-L-lysine (15 μL; ScienCell, Carlsbad, CA, USA) were coated on inserts, incubated at 37 °C for 2 h, washed with DPBS (Merck Life Science, Rome, Italy), and air-dried under sterile circumstances. After being seeded on the basolateral side, CCF-STTG1 cells (3.13 × 10^5^ cells/well) were incubated for 2–3 h at 37 °C in 5% CO_2_. The inserts were then inverted, and any extra media was removed. Fresh culture medium was then added to both compartments: 800 μL to the apical side and 1600 μL to the basolateral side. Plates were incubated for 48 h, after which the astrocyte medium was gently removed to preserve the cell layer. Inserts were then reinverted for subsequent procedures. HBVPs were seeded directly onto the astrocyte layer at a density of 6.25 × 10^4^ cells/well, corresponding to an approximate astrocyte-to-pericyte ratio of 5:1. The plates were incubated for an additional 2–3 h, after which the inserts were returned to their original orientation, excess medium was aspirated, and a 1:1 mixture of astrocyte and pericyte media were added to both compartments. The co-culture was maintained under these conditions until approximately 90% confluency was achieved, typically by day 4.

The apical medium was removed, and HUVECs were seeded onto the collagen-coated apical surface of the inserts at a density of 3 × 10^5^ cells/well. Cells were allowed to adhere for at least 5 h at 37 °C before medium replacement.

After 24 h of HUVEC seeding, TEER was measured using an EVOM2™ voltohmmeter (World Precision Instruments, Hitchin, UK). TEER values were calculated by multiplying the measured resistance (in ohms) by the surface area of the Transwell insert, yielding results in Ω × cm^2^. This setup models key structural and functional features of the human BBB, allowing for the evaluation of barrier integrity and cellular interactions under controlled in vitro conditions.

Treatment and permeability investigations were performed on Transwells^®^ after 7 days of culture [64]. The test compounds were applied to cells for 15 min to 24 h before BBB permeability was measured with a fluorescent marker. Fluorescein (0.04%; Merck Life Science, Rome, Italy) was used to quantify basolateral compartment fluorescence using a spectrophotometer (Infinite 200 Pro MPlex, Tecan, Männedorf, Switzerland) with excitation/emission settings of 490/514 nm. Values were represented as a percentage of the starting chemical that crossed the cell layer, determined using the formula outlined in reference [65]:

Papp = dQ/dt ⇥ 1/m0 ⇥ 1/A ⇥ V Donor (1)

dQ: amount of substance transported (nmol or μg),

dt: incubation time (s),

m0: amount of substrate applied to the donor compartment (nmol or μg),

A: Transwell^®^ membrane surface area (cm^2^),

V Donor: volume of the donor compartment (cm^3^).

The inclusion of cell-free negative controls enabled the elimination of potential interference caused by the Transwell membrane. Each experiment was conducted in triplicate and independently repeated five times.

### 2.5. Cell Viability

Using a conventional method, the MTT in vitro toxicology assay kit (Merck Life Science, Rome, Italy) was employed to measure cell viability following stimulation [52]. Absorbance of all solubilised samples, both treated and controls, was measured at 570 nm and corrected at 690 nm, using a spectrophotometer (Infinite 200 Pro MPlex, Tecan, Männedorf, Switzerland). Results were normalised to the untreated control, set as the 0% reference value of five independent experiments performed in triplicate.

### 2.6. ROS Production

ROS production was assessed by detecting absorbance at 550 nm with a microplate spectrophotometer (Infinite 200 Pro MPlex, Tecan, Männedorf, Switzerland). The cytochrome C reduction was analysed using a conventional methodology [19]. ROS data were obtained from five independent triplicate assays and expressed as mean ± SD (%) of cytochrome C reduction per µg protein, relative to controls.

### 2.7. Claudin 5 Assay Kit

Claudin 5 ELISA Kit (Catalog # MBS7236028, MyBiosource, San Diego, CA, USA) was used to measure the amount of claudin 5 in BBB cell lysates, following the manufacturer’s instructions. The cells were centrifuged at 1500× *g* for 10 min at 4 °C after being lysed with cold phosphate-buffered saline (PBS). Then, 100 μL of each sample was incubated with detection solution A for 45 min at 37 °C, followed by a wash and 45 min with solution B. The substrate solution was then added, and the mixture was incubated in the dark at 37 °C for 20 min. Absorbance was measured at 450 nm using a spectrometer (Infinite 200 Pro MPlex, Tecan, Männedorf, Switzerland) after stopping the reaction using stop solution. A standard curve was used to calculate concentrations from 0.5 to 10 ng/mL. The assay detection limit was 0.1 ng/mL. Results were shown as means (%) ± SD compared to a control of five triple tests

### 2.8. Tricellulin (MARVELD Protein) Assay Kit

Following the manufacturer’s instructions, the Tricellulin ELISA Kit (Catalog # MBS2706976, MyBiosource, San Diego, CA, USA) was used to measure the tricellulin/MARVELD in BBB cell lysates. Centrifuged cells at 1500× *g* for 10 min at 4 °C after lysing them in cold 1× PBS. The wells were incubated at 37 °C for 90 min with 100 μL of each sample. Once the sample was removed, 100 μL of detection solution A was added and incubated at 37 °C for 45 min. After washing, 100 μL of detection solution B was added and incubated for 45 min at 37 °C. Next, 90 μL of substrate solution was added and incubated at 37 °C for 20 min in the dark. After adding 50 μL of stop solution, the reaction was stopped and monitored at 450 nm using a spectrophotometer (Infinite 200 Pro MPlex, Tecan, Männedorf, Switzerland). Concentrations were measured in ng/mL using a standard curve from 0.312 to 20 ng/mL, and the assay detection limit was below 0.125 ng/mL ng/mL. Results were shown as means (%) ± SD compared to a control of five triple tests.

### 2.9. Co-Culture CCF-STTG1/SH-SY5Y In Vitro Model

The method of co-culturing SH-SY5Y cells and astrocytes is reported in the literature [24,57]. Astrocytes were plated at 6 × 10^3^ cells/well in Matrigel-coated 12-well plates with DMEM supplemented with 10% FBS and *N*-2, then incubated overnight at 37 °C. The medium was removed, and SH-SY5Y cells were seeded at 3 × 10^4^ cells/well onto the astrocyte layer in DMEM with 10% FBS. Co-cultures were differentiated over 5 days in medium containing 10 µM retinoic acid and 1% FBS. Only for pPKA ELISA kit, the co-culture was prepared on a 96-well plate (6 × 10^2^ cells/well of astrocytes and 3 × 10^3^ cells/well of SH-SY5Y).

### 2.10. TNFα ELISA Kit

The TNF-α levels were determined in the supernatant of CCF-STTG1/SH-SY5Y co-culture using the human TNF-α ELISA Kit (Catalog # DTA00D, R&D Systems, Inc., Minneapolis, MN, USA) according to the manufacturer’s instructions [66]. The manufacturer’s instructions were followed for reagents, standards, and samples. Assay diluent (50 µL) was applied to each well, followed by 50 µL of standard, control, or sample. For 2 h, plates were sealed and incubated at room temperature. After 5 cycles of washing, 100 µL of substrate solution were added and incubated at room temperature in the dark for 30 min. After stopping the reaction with 100 µL of the stop solution, the absorbance was measured at 450 nm using a microplate reader (Infinite 200 Pro MPlex, Tecan, Männedorf, Switzerland) with correction for wavelengths of 540 or 570 nm. A reference curve that went from 15.6 to 1000 pg/mL was used to compare the concentrations. The assay detection limit was below 6.23 pg/mL ng/mL, and the data are presented as mean ± SD (%) from five independent experiments, each conducted in triplicate.

### 2.11. IL-1β ELISA Kit

The IL-1β levels were determined in the supernatant of the CCF-STTG1/SH-SY5Y co-culture using the Human IL-1β/IL-1f2 ELISA Kit (Catalog # DLB50, R&D Systems, Inc., Minneapolis, MN, USA) according to the manufacturer’s instructions [66]. Reagents, standards, and samples were made per the manufacturer’s protocol. 50 µL of assay diluent and 50 µL of standard, control, or sample were added to the wells, which were then incubated at room temperature for 2 h. 100 µL of substrate was introduced after five washes and incubated in the absence of light for 30 min. A spectrometer (Infinite 200 Pro MPlex, Tecan, Männedorf, Switzerland) was used to measure absorbance at 450 nm with wavelength correction at 540/570 nm after stopping the reaction with 100 µL stop solution. A standard curve ranging from 3.9 to 250 pg/mL was used to calculate concentrations. The assay detection limit was 1 pg/mL ng/mL, and the data are presented as mean ± SD (%) from five independent experiments, each conducted in triplicate of five independent experiments performed in triplicate.

### 2.12. FAAH Assay Kit

FAAH activity was assessed on supernatants of CCF-STTG1/SH-SY5Y co-culture, as reported in the literature [67]. Total sample preparations were tested for enzymatic activity using two centrifugations at 30,000× *g* for 30 min. The assays used 0.5–1 mg of tissue, incubated at 37 °C for 30 min in 200 µL of Tris-EDTA buffer (pH 7.4) with 1, 5, or 20 µM [^3^H]-AEA (American Radiolabelled Chemicals, Maryland Heights, MO, USA). Add 400 µL of 8% (*w*/*v*) activated charcoal in 0.5 M HCl to inhibit reactions, then centrifuge at 13,000× *g* for 5 min. [^3^H]-Ethanolamine levels were measured within the supernatant using a plate reader (Infinite 200 Pro MPlex, Tecan, Männedorf, Switzerland) at 360/460 nm. Data are presented as mean ± SD (%) from five duplicate trials compared to untreated controls.

### 2.13. NAAA ELISA Kit

The human NAAA ELISA Kit (Catalog # MBS2021396, MyBiosource, San Diego, CA, USA) provided a microplate pre-coated with an antibody specific to NAAA. Wells pre-coated with biotinylated anti-NAAA antibodies received standards or CCF-STTG1/SH-SY5Y co-culture lysates. TMB substrate was added after incubating Avidin-HRP conjugate. Colourimetric reaction in NAAA-containing wells was inhibited by sulfuric acid. Using a plate reader (Infinite 200 Pro MPlex, Tecan, Männedorf, Switzerland), absorbance was measured at 450 ± 10 nm, and NAAA concentrations were obtained by comparing O.D. values to a standard curve (0.312–20 ng/mL). The assay detection limit was 0.115 ng/mL. The results were expressed as means ± SD (%) compared to the control of five independent experiments performed in triplicate.

### 2.14. NAAA Activity

To evaluate NAAA activity, in vitro click chemistry Activity-Based Protein Profiling (CC-ABPP) on CCF-STTG1/SH-SY5Y co-culture lysates prepared in 4 volumes of PBS/Sucrose 0.32 M at pH 7.4 supplemented with protease inhibitor cocktail (Merck Life Science, Rome, Italy) and incubated with ARN726 (40 μM, Merck, Darmstadt, Germany) or ARN077 (40 μM, Merck, Darmstadt, Germany) following a standard protocol was performed [68]. The sample was centrifuged at 800× *g* for 20 min at 4 °C, and protein content was assessed using the BCA assay (Thermo Fisher Scientific, Waltham, MA, USA). The reaction was mixed with vortex shaking and incubated for 1 h at 25 °C. After the reaction time, the samples were analysed using a protein blot. Specifically, 1× SDS loading buffer was added to the samples, and ABP-labelled proteome enriched on streptavidin beads, 1/10 of the total enrichment was first eluted from the beads by adding two volumes of a solution of 6 M urea, 2 M thiourea, 6 mM biotin and 2% SDS, 15 min RT followed by 15 min at 95 °C, 5 then 1× SDS loading buffer was added. Using SDS-PAGE, the samples were resolved and put on nitrocellulose sheets that had been blocked with 5% BSA/PBS/0.1% Tween 20. The specific monoclonal mouse anti-HASAHL antibody (MAB4494, R&D Systems) was used to detect the expression of NAAA. A species-specific peroxidase-conjugated secondary antibody was used to detect the reaction of primary antibodies. At the same time, streptavidin-peroxidase conjugate (Merck Life Science, Rome, Italy) was added to detect biotinylated samples. Images were acquired with the Chemidoc instrument (BioRad, Hercules, CA, USA) after adding a chemiluminescent substrate (ECL, BioRad, Hercules, CA, USA). Data are presented as mean ± SD (%) from five independent experiments, each conducted in triplicate.

### 2.15. AG ELISA Kit

The levels of 2-AG were measured using a specific 2-AG ELISA Kit (Catalog # CEO443Ge, Cloud-Clone Corp., Katy, TX, USA) in the supernatant of CCF-STTG1/SH-SY5Y co-cultures. In summary, 50 µL of sample and 50 µL of detection reagent A were added to each well. The plate was then incubated for one hour at 37 °C, following a process of shaking and stirring. Following the conclusion of the incubation period, the wells underwent a washing process on three separate occasions. Thereafter, 100 µL of detection reagent B was introduced to the wells. Following a 30 min incubation at 37 °C, the wells were subjected to a washing step consisting of five cycles. Subsequently, 90 µL of substrate solution was added to each well, and the plate was incubated at 37 °C for 10–20 min. After the incubation period, 50 µL of stop solution was added to each well, and the plate was analysed at a wavelength of 450 nm using a spectrophotometer (Infinite 200 Pro MPlex, Tecan, Männedorf, Switzerland). The concentration was expressed in ng/mL by comparing the data with the calibration curve (3.70–300 ng/mL). The minimum detectable dose of this kit is less than 1.44 ng/mL. The results were represented as mean ± SD compared to the control (untreated cells, 0% line) of five independent experiments performed in triplicate.

### 2.16. AEA ELISA Kit

The levels of AEA were measured by human Anandamide ELISA Kit (Catalog # MBS167217, MyBiosource, San Diego, CA, USA) in the supernatant of CCF-STTG1/SH-SY5Y co-culture. To each well, add 40 µL of samples, 10 µL of anti-AEA antibody, and 50 µL of streptavidin-HRP. Five washes were performed on wells after 1 h of incubation at 37 °C. Following the addition of 50 µL of substrates A and B, the plate was incubated for 10 min at 37 °C in the dark. To quantify absorbance, the reaction was stopped with 50 µL of stop solution and measured at 450 nm (Infinite 200 Pro MPlex, Tecan, Switzerland). AEA levels (0.05–20 ng/mL) were quantified using the standard curve, with a sensitivity of <0.022 ng/mL. Data reported as mean ± SD vs. untreated control.

### 2.17. CB2R ELISA Kit

The CB2R ELISA Kit (Catalog # LS-F49345-1, LSBio, Newark, CA, USA) was used in CCF-STTG1/SH-SY5Y co-culture supernatants, following the manufacturer’s instructions [69]. 100 µL of sample was incubated at 37 °C for 90 min in each well. Preparation involved washing wells twice and incubating with 100 µL of biotin-conjugated antibody at 37 °C for 60 min. Add 100 µL of SABC working solution after three washes and incubate for 30 min. After five washes, 90 µL of TMB substrate was added to the wells. stop solution (50 µL) was added after 10–20 min, and absorbance was measured at 450 nm (Infinite 200 Pro MPlex, Tecan, Switzerland). A standard curve (78–5000 pg/mL) was used to determine analyte concentrations, with a sensitivity of 78 pg/mL. Data from five independent triplicate experiments were presented as mean ± SD (%) compared to the untreated control.

### 2.18. PPARα Assay Kit

PPARα was measured by the PPAR alpha Transcription Factor Assay Kit (Catalog # ab133107, Abcam, Cambridge, UK) on nuclear extracts, following the manufacturer’s instructions. The nuclear extraction buffer with protease and phosphatase inhibitors was used to lyse the CCF-STTG1/SH-SY5Y co-culture. The mixture was subsequently centrifuged at 14,000× *g* for 10 min at 4 °C. The transcription factor binding assay buffer was added to the plate with nuclear-containing supernatant. After incubation with PPARα primary and secondary antibodies, absorbance was measured at 450 nm using an Infinite 200 Pro MPlex (Tecan, Männedorf, Switzerland). Data from five independent triplicate experiments are presented as mean (%) ± SD versus control [52].

### 2.19. Phospho-TRPV1 ELISA Kit

The amounts of pTRPV1 in CCF-STTG1/SH-SY5Y co-culture lysates were measured by ELISA assay following the guidance reported in the literature [70]. After coating a 96-well plate overnight at 4 °C with 5 μg/mL of anti-phospho-TRPV1 antibody, it was blocked with 1% Block Ace at room temperature for 2 h. Then, 50 μL of protein lysate was added and incubated overnight at 4 °C after the PBST washes. The wells were treated with 50 μL of streptavidin-poly-HRP (500 ng/mL) and biotinylated anti-TRPV1 antibody (500 ng/mL) for 2 h at room temperature. After the PBST wash, 50 μL of TMB substrate was mixed in. Stopping the reaction with 0.5 N sulfuric acid, the absorbance was measured at 450 nm (Infinite 200 Pro MPlex, Tecan, Männedorf, Switzerland). Data from five independent triplicate assays are presented as mean ± SD (%) relative to the control.

### 2.20. Phospho-PKA α/β CAT (Thr197) Colorimetric Cell-Based ELISA Kit

The pPKA α/β CAT (Thr197) Colorimetric Cell-Based ELISA Kit (Catalog # EKC2107, Boster Biological Technology, Pleasanton, CA, USA) was used on CCF-STTG1/SH-SY5Y co-culture plated in a 96-well as indicated in Section 2.9. Stimulated cells were fixed with 4% formaldehyde for 20 min at room temperature, followed by three washes with 1× wash buffer. Afterwards, additional washes were conducted, followed by a 20 min quenching step. Then 200 µL of blocking buffer was applied for 1 h, washed, and added 50 µL of anti-PKA α/β CAT primary antibody for overnight incubation at 4 °C. Following this, the wells were washed and incubated with 50 µL of HRP-conjugated secondary antibody at room temperature for 90 min. After washing, 50 μL of substrate was added, incubated for 30 min in the dark, and stopped with 50 μL of stop solution. Measurements of absorbance were conducted at 450 nm (Infinite 200 Pro MPlex instrument from Tecan, Männedorf, Switzerland). Five independent experiments, each conducted in triplicate, yielded results expressed as mean ± SD (%) relative to untreated controls.

### 2.21. Western Blot

The co-culture CCF-STTG1/SH-SY5Y was lysed, following the standard protocol [52], in ice with complete tablet buffer (Roche, Basilea, Svizzera) supplemented with 1 mM phenylmethanesulfonyl fluoride (PMSF), 2 mM sodium orthovanadate (Na_3_VO_4_) (Merck Life Science, Rome, Italy), a 1:50 mix of phosphatase inhibitor cocktail (Merck Life Science, Rome, Italy), and a 1:200 mix of protease inhibitor cocktail (Merck Life Science, Rome, Italy). Then, 35 μg of protein from each sample was resolved on a 10% SDS-PAGE gel. The polyvinylidene difluoride membranes (PVDF, GE, Healthcare Europe Gmbh, Ruma, Serbia) were incubated overnight at 4 °C with the specific primary antibody anti-CB2 (1:500; Santa Cruz, CA, USA). Anti-β-actin (Merck Life Science, Rome, Italy) detection was used to normalise and verify protein expression. Data from five independent triplicate experiments were presented as mean ± SD (%) compared to the untreated control.

### 2.22. Statistical Analysis

Data are the mean ± SD of at least five biological replicates, analysed in triplicate. To emphasise intergroup differences, the control was set to 0%, and the values were normalised to the control by averaging triplicates and expressing the results as a percentage. Using GraphPad Prism 10.2.3 (GraphPad Software, La Jolla, CA, USA), one-way ANOVA with Bonferroni post hoc test, or the Mann–Whitney U test was used for statistical analysis. Significance was defined as *p*-value < 0.05.

## 3. Results

### 3.1. Evaluation of BBB Permeability and Integrity in an In Vitro Tri-Culture Model

**Figure 2:**
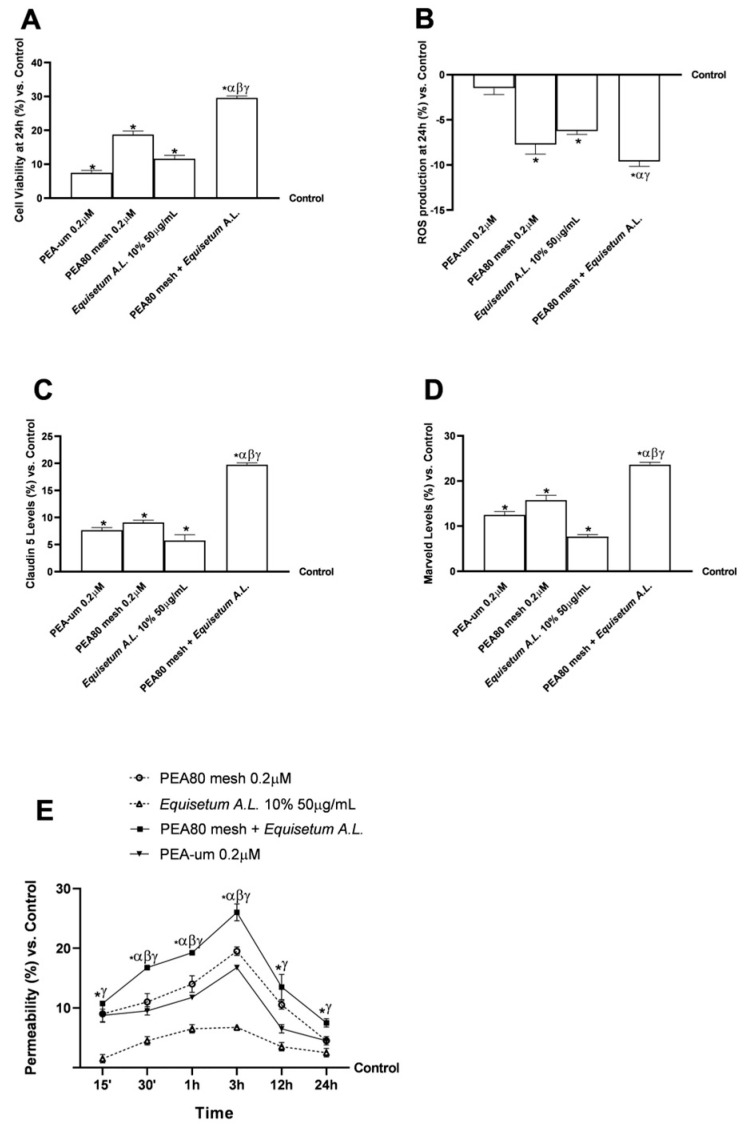
Analysis of a 3D tri-culture model of BBB after 24 h treatment with the individual agents and combination under consideration. (**A**) Cell viability results at 24 h through MTT assay; (**B**) oxidative stress results at 24 h in terms of ROS production by colorimetric method; (**C**,**D**) levels of TJ at 24 h obtained by ELISA kit; and (**E**) sample permeability to predict the bioavailability in the brain after crossing the BBB through fluorescent probe. Data are expressed as means ± SD (%) of increased values derived from five independent experiments performed in triplicate, normalised to control values (0% line). * *p* < 0.05 vs. control; α *p* < 0.05 vs. PEA-um 0.2 μM; β *p* < 0.05 vs. PEA80mesh 0.2 μM; γ *p* < 0.05 vs. *Equisetum A.L.* 10% 50 μg/mL.

Investigations have been conducted using an in vitro 3D model that simulates the state of functionality and complexity of the BBB in vivo to assess the cytotoxicity of the test substances and collect important absorption data. This is crucial, as the BBB is responsible for maintaining adequate conditions in the integral environment of the nervous system. Figure 2A shows that all tested drugs significantly increased cell viability at 24 h compared to the control (*p* < 0.05). PEA80mesh 0.2 μM + *Equisetum A.L.* 50 μg/mL demonstrated better cell viability with a 37% difference at PEA 80 mesh 0.2 μM, 60% compared to *Equisetum A.L.* 50 μg/mL, and 75% compared to PEA ultramicronized (PEA-um) 0.2 μM. Additionally, tests were conducted to assess the rate of reactive oxygen species (ROS) production after 24 h of treatment with the test samples. Figure 2B shows that all investigated substances decreased the production of ROS compared to the control (*p* < 0.05) and maintained levels within physiological limits. The integrity of the in vitro BBB was then examined and confirmed by TJ analysis. In this context, the activities of Claudin-5 and Marveld were characterised to determine any changes following treatment. The first is a key component of the TJ filament in brain endothelial cells. It is responsible for the selective reduction in ion permeability [71]. On the other hand, the second provides the maintenance of the barrier to the passage of the macromolecule and allows the release of the effector downstream [72]. As reported in Figure 2C, data from evaluating claudin-5 levels on the BBB showed a significant effect after stimulation with the combination examined than the control (*p* < 0.05). For all individual agents, the combination of PEA80mesh 0.2 μM and *Equisetum A.L*. 50 μg/mL has promoted an increase of 10% vs. PEA80mesh 0.2 μM and PEA-um 0.2 μM and 15% vs. *Equisetum A.L.* 50 μg/mL. The same effect was observed on the Marveld levels (Figure 2D): the combination had a more significant impact compared to the control and the single agents (*p* < 0.05). Subsequent investigations were conducted on samples for 15′–30′ and 1–3–12–24 h. As demonstrated in Figure 2E, the combination of PEA80mesh 0.2 μM and *Equisetum A.L.* 50 μg/mL exhibited time-dependent absorption from 30 min to 24 h compared to the untreated control (*p* < 0.05). This fluorescence analysis in the basolateral environment assesses the substances that cross the BBB and reach the brain. The BBB data specifically showed that the chemicals may travel through it. Specifically, the combination of PEA80mesh 0.2 μM and *Equisetum A.L.* 50 μg/mL demonstrated good absorption at the BBB level, with a peak absorption at 3 h, out of the two types of PEAs that were evaluated. These three samples exhibited noteworthy values for every time frame compared to the control, and only *Equisetum A.L.* 50 μg/mL recorded permeability data below 10% around the permeability peak (4 h). Throughout the analysis period, more products crossed the BBB than single substances (*p* < 0.05). This effect was most noticeable around 3 h, during which the combination of PEA80mesh 0.2 μM and *Equisetum A.L*. 50 μg/mL exhibited an absorption of 25% (*p* < 0.05), suggesting that the combination is responsible for the increased permeability.

### 3.2. Analysis of Biological Effects of the Combination of PEA80mesh + Equisetum A.L. on Co-Culture CCF-STTG1/SH-SY5Y After Crossing the BBB

**Figure 3:**
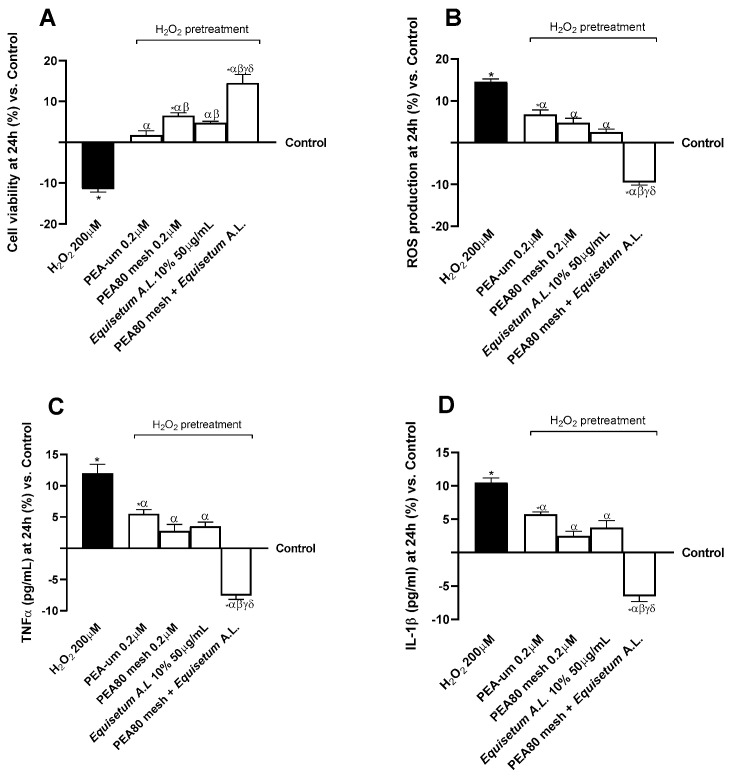
Analysis of the biological effects of test samples in terms of cytotoxicity and inflammation after treatment for 24 h on CCF-STTG1/SH-SY5Y co-culture. (**A**) Cell viability results through MTT assay; (**B**) oxidative stress results in terms of ROS production by colorimetric method; n (**C**) levels of TNFα (pg/mL) obtained by ELISA kit; and (**D**) levels of IL-1β (pg/mL) obtained by ELISA kit. Data are expressed as means ± SD (%) of increased values derived from five independent experiments performed in triplicate, normalised to control values (0% line). * *p* < 0.05 vs. control; α *p* < 0.05 vs. H_2_O_2_ 200μM; β *p* < 0.05 vs. PEA-um 0.2 μM; γ *p* < 0.05 vs. PEA80mesh 0.2 μM; δ *p* < 0.05 0.05 vs. *Equisetum A.L.* 10% 50 μg/mL.

Following the analysis conducted on the in vitro model of the BBB, the supernatant of the basolateral compartment of the Transwell^®^ system, containing the metabolite and biological material that crossed the BBB, was used as a stimulus for 24 h in the CCF-STTG1/SH-SY5Y co-culture model. Pre-treatment with H_2_O_2_ 200 μM as indicated in the literature [63] was essential to induce neuroinflammation in vitro. At this stage, the safety analyses of the test samples were conducted, investigating the maintenance of cell viability and the levels of ROS produced, as well as biological markers directly related to the initiation of the inflammatory response. As can be seen in Figure 3, for all examined parameters H_2_O_2_ 200 μM demonstrated a neurotoxic, pro-oxidant, and inflammatory effect by reducing cell viability and targeting a neuroinflammatory context with statistically significant data compared to the control (*p* < 0.05). All test samples demonstrated counteracting the negative action induced by H_2_O_2_ 200 μM (*p* < 0.05), with more significant data from the combination of PEA80mesh + *Equisetum A.L.* In detail, as shown in Figure 3A, among the single agents PEA80mesh 0.2 μm and *Equisetum A.L.* 50 μg/mL yielded more significant results than PEA-um 0.2 μm alone, with percentage differences of 65% and 52%, respectively. Treatment with the combination of PEA80mesh + *Equisetum A.L.* enhanced the effect of the single agents with a 1.8-fold increase in cell viability compared to H_2_O_2_ 200 μM, 74% compared to PEA-um 0.2 μM, 55% compared to PEA80mesh 0.2 μM, and 67% compared to *Equisetum A.L.* 50 μg/mL. At the same time, the combination of PEA80mesh + *Equisetum A.L.* amplified the antioxidant effect (Figure 3B) of the individual constituents with a 2.5-fold reduction in ROS production compared to H_2_O_2_ 200μM, a 1.7-fold reduction compared to PEA-um 0.2 μM, a 1.5-fold reduction compared to PEA80mesh 0.2 μM, and a 73% reduction compared to *Equisetum A.L.* 50 μg/mL. Moreover, even in the inflammatory context (Figure 3C,D), the combination examined contributed the most significant results in modulating the induced neurotoxic effect. The mean percentage reduction in pg/mL of pro-inflammatory markers (TNFα and IL-1β) was 2.6-fold compared to pretreatment with H_2_O_2_ 200 μM, by 1.9-fold compared to PEA-um 0.2 μM, by 1.4-fold compared to PEA80mesh 0.2 μM, and by 1.7-fold compared to *Equisetum A.L.* 50 μg/mL.

### 3.3. Assessment of the Effects of the Combination of PEA80mesh + Equisetum A.L. on Biological Mechanisms Linked to Nociception on Co-Culture CCF-STTG1/SH-SY5Y

**Figure 4:**
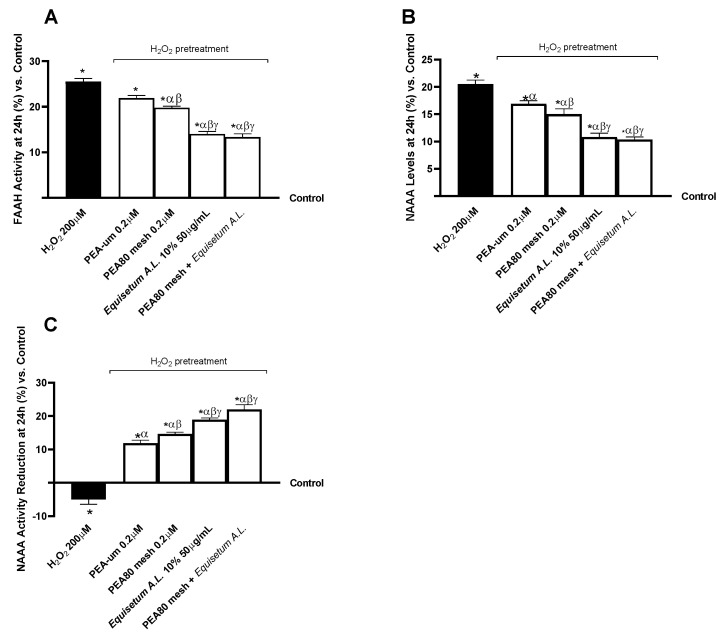
Analysis of biomarkers correlated with the intracellular metabolism of PEA on CCF-STTG1/SH-SY5Y co-culture after treatment for 24 h with test samples. (**A**) FAAH activity; (**B**) NAAA levels, obtained by ELISA kit; and (**C**) NAAA activity reduction with inhibitory probe. The results are expressed as mean ± SD (%) of 5 normalised biological replicates performed in triplicate vs. control (0% line). * *p* < 0.05 vs. control; α *p* < 0.05 vs. H_2_O_2_; β *p* < 0.05 vs. PEA-um 0.2μM; γ *p* < 0.05 vs. PEA80mesh 0.2 μM.

In this experimental part, this study focuses on the critical role of PEA metabolism in initiating the nociception and pain perception signalling pathway in the CNS. In this context, the analysis of the activity of the FAAH, the NAAA, as well as its levels, was crucial. As performed in previous experiments, the CCF-STTG1/SH-SY5Y co-culture was pre-treated for 30 min with H_2_O_2_ 200 µM before proceeding with the 24 h treatment of test samples. In detail, Figure 4A shows that a half-hour pretreatment with H_2_O_2_ 200 µM increased intracellular FAAH activity by 25.50% compared to the untreated control (*p* < 0.05). The individual PEA favoured a decrease in its activity of 22% and 19.80%, respectively, which proved to be greater after treatment with only *Equisetum A.L.* 50 μg/mL and with the PEA80mesh 0.2 μM + *Equisetum A.L*. 50 μg/mL formulation. PEA80mesh 0.2 μM + *Equisetum A.L.* 50 μg/mL and *Equisetum A.L.* 50 μg/mL showed a similar and substantial benefit to PEAs (*p* < 0.05). Regarding NAAA levels revealed by the ELISA kit (Figure 4B), PEA80mesh 0.2 μM + *Equisetum A.L.* 50 μg/mL was shown to decrease intracellular levels of NAAA by about 98% than the H_2_O_2_ 200 µM (*p* < 0.05). Furthermore, PEA80mesh 0.2 μM + *Equisetum A.L.* 50 μg/mL decreased by about 63% than PEA-um 0.2 μM alone and 45% than PEA80mesh 0.2 μM (*p* < 0.05). *Equisetum A.L.* 50 μg/mL showed a similar effect to PEA80mesh 0.2 μM + *Equisetum A.L*. 50 μg/mL. In addition, after performing an enzyme activity analysis of NAAA (Figure 4C), it was shown that PEA80mesh 0.2 μM + *Equisetum A.L.* 50 μg/mL gave the most relevant results regarding NAAA activity reduction after using an enzyme activity inhibitor probe. The effect promoted by PEA80mesh 0.2 μM + *Equisetum A.L.* 50 μg/mL showed a 7.5-fold percentage increase in reduction on NAAA activity over H_2_O_2_ 200 µM, 2.1-fold over PEA-um, and 50% over PEA80mesh (*p* < 0.05). *Equisetum A.L.* 50 μg/mL demonstrated a similar effect to the combination, which is significant compared to PEA alone (*p* < 0.05), but slightly lower and not statistically significant compared to PEA80mesh 0.2 μM + *Equisetum A.L.* 50 μg/mL.

**Figure 5:**
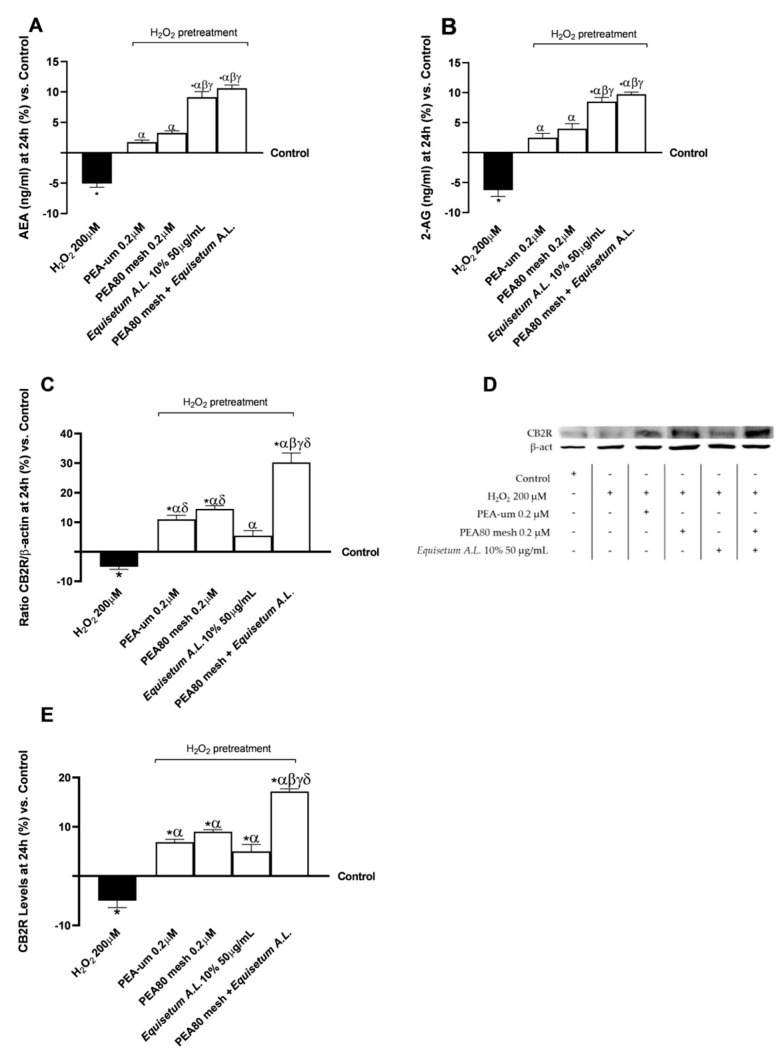
Analysis of biomarkers correlated with the endocannabinoid system on CCF-STTG1/SH-SY5Y co-culture after treatment for 24 h with test samples. (**A**) AEA levels obtained by ELISA kit; (**B**) 2-AG levels obtained by ELISA kit; (**C**) CB2R densitometric analysis after the Western blot, which is reported as an example image in (**D**); and (**E**) CB2R levels obtained by ELISA kit. The results are expressed as mean ± SD (%) of 5 normalised biological replicates performed in triplicate vs. control (0% line). * *p* < 0.05 vs. control; α *p* < 0.05 vs. H_2_O_2_; β *p* < 0.05 vs. PEA-um 0.2μM; γ *p* < 0.05 vs. PEA80mesh 0.2 μM; δ *p* < 0.05 vs. *Equisetum A.L.* 10% 50 μg/mL.

After obtaining the results on the modulation of the test samples on both activity and enzyme levels crucial to the metabolism of PEA and the endocannabinoid system, the levels of two key markers of the endocannabinoid system (AEA and 2-AG) were investigated. As can be seen in Figure 5A,B, pretreatment with H_2_O_2_ 200 µM contributed to a reduction in ng/mL levels of both AEA and 2-AG compared to the control condition, consistent with the results obtained in the previous series of analyses (Figure 4). All test samples counteracted the negative action promoted by H_2_O_2_ 200 µM by favouring an increase in the levels of both examined markers after 24 h of treatment (*p* < 0.05). More significant effects were observed after stimulation with a combination of PEA80mesh 0.2 μM + *Equisetum A.L.* 50 μg/mL, which yielded similar data to those obtained with *Equisetum A.L.* 50 μg/mL alone, compared to the two forms of PEA examined (*p* < 0.05). In detail, PEA80mesh 0.2 μM + *Equisetum A.L.* 50 μg/mL increased AEA levels by 1.5-fold compared to H_2_O_2_ 200 µM, 84% compared to PEA-um 0.2 μM, 71% compared to PEA80mesh 0.2 μM, and 14% compared to *Equisetum A.L.* 50 μg/mL. At the same time, PEA80mesh 0.2 μM + *Equisetum A.L.* 50 μg/mL increased AEA levels 1.6-fold compared to H_2_O_2_ 200 µM, 74% compared to PEA-um 0.2 μM, 60% compared to PEA80mesh 0.2 μM, 13% compared to *Equisetum A.L.* 50 μg/mL.

At this phase, analyses were carried out on the modulation of CB2R levels by the test samples, both in terms of densitometric analysis (Figure 5C,D) and by means of specific ELISA kits. Regarding the densitometric analysis following the Western blot, pretreatment with H_2_O_2_ 200 µM induced a reduction in the presence of CB2R in the lysates of CCF-STTG1/SH-SY5Y co-culture compared to the control condition (*p* < 0.05). All the individual agents tested, and the combination contributed to counteracting the negative action of H_2_O_2_ 200 µM by increasing CB2R densitometric analysis data (*p* < 0.05), with data also higher than the control (*p* < 0.05; except *Equisetum A.L.* 50 μg/mL). PEA80mesh 0.2 μM + *Equisetum A.L.* 50 μg/mL increased CB2R densitometry data by 7-fold compared to H_2_O_2_ 200 µM, 64% compared to PEA-um 0.2 μM, 52% compared to PEA80mesh 0.2 μM, 82% compared to *Equisetum A.L.* 50 μg/mL. Data were also confirmed after performing the specific ELISA kit (Figure 5E) for the determination of CB2R levels. PEA80mesh 0.2 μM + *Equisetum A.L.* 50 μg/mL increased CB2R levels by 4.5-fold compared to H_2_O_2_ 200 µM, 60% compared to PEA-um 0.2 μM, 48% compared to PEA80mesh 0.2 μM, 71% compared to *Equisetum A.L.* 50 μg/mL.

**Figure 6:**
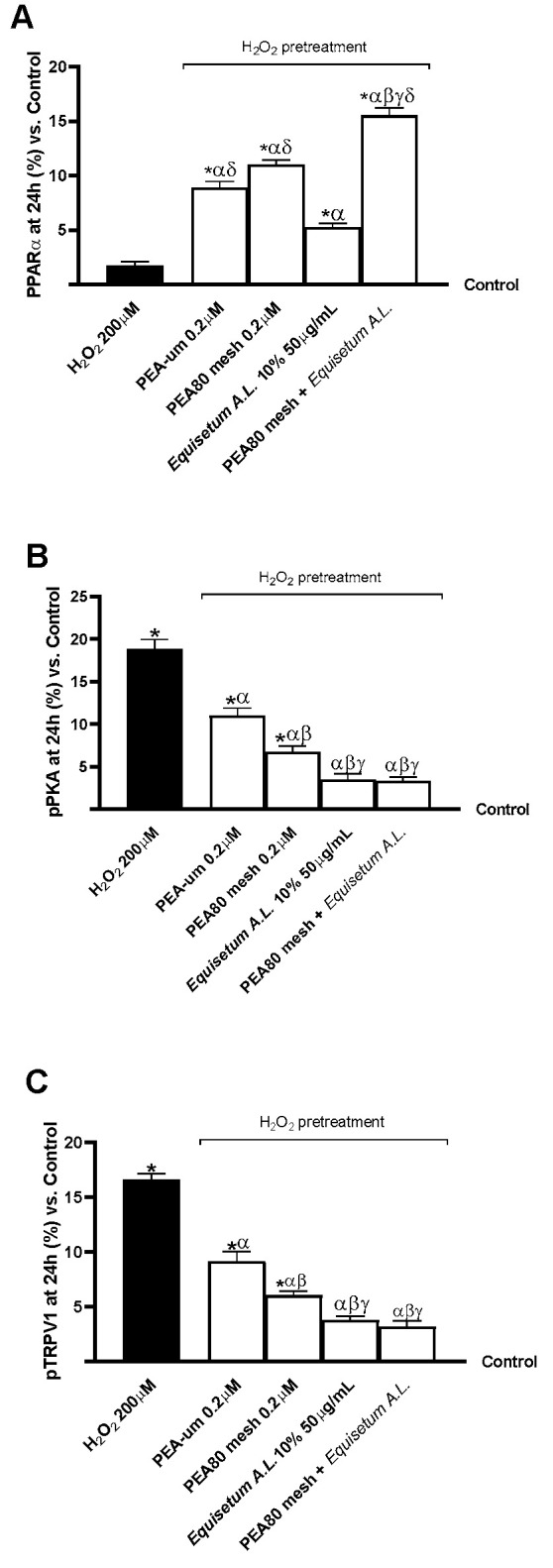
Analysis of biomarkers linked with the analgesic response on CCF-STTG1/SH-SY5Y co-culture after treatment for 24 h with test samples. (**A**) PPARα activity; (**B**) pPKA expression; and (**C**) pTRPV1 expression. The results are expressed as mean ± SD (%) of 5 normalised biological replicates performed in triplicate vs. control (0% line). * *p* < 0.05 vs. control; α *p* < 0.05 vs. H_2_O_2_; β *p* < 0.05 vs. PEA-um 0.2 μM; γ *p* < 0.05 vs. PEA80mesh 0.2 μM; δ *p* < 0.05 vs. *Equisetum A.L.* 50 μg/mL.

Lastly, biological markers directly related to the nociceptive signalling pathway and its analgesic influence were examined. As shown in Figure 6, the expression of some markers, including PPARα, pPKA, and pTRPV1, was reported using ELISA kits. After being pretreated for 24 h, all the samples under study had considerably higher PPARα levels than 200 µM H_2_O_2_ and the control group (*p* < 0.05). PEA80mesh 0.2 μM + *Equisetum A.L.* 50 μg/mL demonstrated a significant effect in terms of increased PPARα levels compared to other samples, differing by 77% from 200 µM H_2_O_2_, 42.50% than PEA-um 0.2 μM, 29% than PEA80mesh 0.2 μM, and almost 2-fold than *Equisetum A.L.* 50 μg/mL (Figure 6A, *p* < 0.05). The pPKA protein’s expression was also examined in this setting, as shown in Figure 6B. pPKA expression was significantly lower in all samples when compared to 200 µM H_2_O_2_ (*p* < 0.05). The most significant reduction in protein expressions was also observed in the presence of *Equisetum A.L.* 50 μg/mL and PEA80mesh 0.2 μM + *Equisetum A.L.* 50 μg/mL, with differences of almost 4.5 times when compared to 200 µM H_2_O_2_, more than 2 times when compared to PEA-um 0.2 μM, and 1.05 times when compared to PEA80mesh 0.2 μM (*p* < 0.05). Comparable outcomes for the study of pTRPV1 expressions can be found in Figure 6C. pTRPV1 expressions were significantly lower in all samples when compared to 200 µM H_2_O_2_ (*p* < 0.05). The most significant decrease in protein expression was additionally observed in *Equisetum A.L.* 50 μg/mL and PEA80mesh 0.2 μM + *Equisetum A.L.* 50 μg/mL, with a percentage difference of almost 4-fold when compared to 200 µM H_2_O_2_, over 1.5-fold when compared to PEA-um 0.2 μM, and 76.50% when compared to PEA80mesh 0.2 μM (*p* < 0.05).

## 4. Discussion

The BBB regulates nutrient transport, restricts the passage of harmful substances, and protects neural integrity by selectively regulating the exchange of substances between the central nervous system and peripheral circulation [73]. BBB dysfunction is linked to stroke, epilepsy, MS, and Parkinson’s and Alzheimer’s disease [74]. The first phase used a tri-culture Transwell^®^ model of endothelial, pericyte, and astrocytes to verify BBB integrity. This method is indicative of the cellular complexity of the native BBB, which is maintained by densely connected brain endothelial cells, pericytes, astrocytes, microglia, and neurons that collectively guard its structural and functional characteristics [75,76].

In this context, the 3D in vitro model of BBB was used to characterise not only the possible cytotoxic effects but also the permeation state of the samples examined. The data obtained showed that all the samples analysed induced no cytotoxic effect, with optimal data in terms of TJ levels, confirming the proper maintenance of BBB integrity. Indeed, all samples have demonstrated the ability to cross the BBB without compromising its integrity and functionality. Indeed, the analysis of the TJ levels, fundamental in maintaining the BBB’s functionality, such as claudina-5 and marveld, allowed us to demonstrate the absence of damage induced by the samples. Claudin 5 is a key component of TJ filament in cerebral endothelial cells and is responsible for the selective reduction in ionic permeability. At the same time, tricellulin, also known as marveld, is crucial for the BBB’s selective permeability and maintaining cellular polarisation. It interacts with tight junctions, such as claudin proteins, to strengthen the barrier function [77]. Their deficiency causes BBB’s selective dimensional loosening [71,78]. These data were suggested by previous findings about the integrity of the intestinal barrier and the effectiveness observed on the peripheral nervous system [52]. They exhibited a similar absorption pattern at the BBB level during the investigated period, with a peak around 3 h of stimulation. The two forms of PEA showed good permeation through the BBB, with higher data from PEA80mesh than the PEA-um, highlighting that this parameter depends on the structure and shape of the substance. Indeed, this was hypothetical; the PEA compound was able to cross the BBB and then accumulate later in the hypothalamus, pituitary, brain stem, cerebellum, and cerebral cortex [11]. PEA80mesh 0.2 μM + *Equisetum A.L*. 50 μg/mL allowed to improve not only the absorption through the BBB of PEA80mesh but also the absorption kinetics of *Equisetum A.L.* 50 μg/mL (*p* < 0.05). PEA80mesh 0.2 μM + *Equisetum A.L.* 50 μg/mL has increased the absorption rate of PEA80mesh 0.2 μM by 33% around 3 h of treatment and 2.85-fold regarding *Equisetum A.L.* 50 μg/mL. Optimal in vitro results on permeability through the BBB were also obtained in our other recent research, where the combination of different natural extracts recorded peak uptake around 12 h, maintaining their effect up to 24 h after stimulation [4].

Following the passage and metabolization at the level of the BBB model in vitro, it was possible to demonstrate maintenance of the bioactive effect of the test samples at the level of the CNS. The co-culture of SH-SY5Y cells and astrocytes have demonstrated excellent potential for neuroprotection studies in the literature [57,79]. Indeed, glial cells secrete neurotrophic factors that can modulate neuroprotective mechanisms in the presence of neurotoxicity [80]. Genes related to neuronal damage were highly expressed in co-culture conditions compared to monoculture, suggesting excellent potential for transposition to the human brain environment [57]. Furthermore, the co-culture demonstrated anti-inflammatory and phagocytic responses to pathogens as well as protective efficacy against heavy metal-induced neurotoxicity by inhibiting the decline in glutathione levels, an antioxidant [81]. According to the scientific evidence discussed earlier, the selected neuronal co-culture enabled the characterisation of the protective action of the test sample in the context of H_2_O_2_-induced neurotoxicity, contributing to the induction of oxidative stress and the subsequent triggering of the inflammatory response. H_2_O_2_-induced oxidative stress, is a significant factor in the development of astrogliosis and inflammation associated with astrocytes. ROS can stimulate many signalling pathways associated with inflammation in astrocytes and can facilitate the release of inflammatory factors [82]. Research has demonstrated that ROS originating from mitochondria can trigger the inflammatory cascade in astrocytes by encouraging the cleavage of pro-caspase-1. Subsequently, cleaved caspase-1 can cleave IL-1β and IL-18 precursors, facilitating the release of IL-1β and IL-18. Astrogliosis can result in glial scarring in advanced stages of CNS damage, which is considered the primary physical obstacle preventing neuronal axonal regrowth [83]. All individual agents proved to be significantly responsive in limiting the neurotoxic action induced by H_2_O_2_ 200 μM, as evidenced by improvements in cell viability and reductions in the production of ROS and pro-inflammatory cytokines. The effect of PEA80mesh at 0.2 μM was most amplified by the combination with *Equisetum A.L.* at 50 μg/mL. This is strongly supported by the co-operative action of the bioactive molecules associated with the extract documented in the literature, such as flavonoids (e.g., quercetin), phenolic acids (caffeic, ferulic, *p*-coumaric), and phytosterols (β-sitosterol), with good neuro-protective, anti-inflammatory and antioxidant properties, which is also critical [84,85]. These bioactive properties have helped to improve the anti-inflammatory but especially antioxidant profile of PEA, which has been described as a limiting factor of the substance that can be enhanced by association with natural elements or extracts with antioxidant potential. This aspect was also supported by the CB2R analysis. First, this provided confirmatory data on the correct crossing of substances across the BBB pattern and their subsequent impact on the CNS, specifically regarding analgesia. As has also been shown in numerous studies, PEA has an affinity for CB2R that contributes to pain control [36,86,87,88]. Indeed, both forms of PEA showed a significant effect in modulating the presence of CB2R at the level of the CCF-STTG1/SH-SY5Y co-culture, not only in comparison to control but also in comparison to H_2_O_2_-induced damage. The different cut-off present in the PEA80mesh was shown to enhance this interaction and related mechanisms, such as anti-inflammatory action, confirming what was obtained in a previous study [36].

In nociception, CB2R is linked to the endocannabinoid system, which regulates immune function, energy balance, cognitive processes, and neuronal signalling [88]. The combination of CB2R and endocannabinoids contributes to the immunosuppressive and anti-inflammatory effect by modulating the release of cytokines [89]. In addition, CB2R, as also confirmed in our co-culture model, are expressed at the level of the CNS, in microglia and on astrocytes and, when stimulated, they reduce the pro-inflammatory forms of these cells, resulting in a reduction in chronic pain and nociception [90]. Recent studies have focused on modulating endocannabinoid levels by inhibiting FAAH [91,92]. Well-known is PEA in this field [93] but also plant extracts and purified chemicals are examples of natural substances that can inhibit FAAH and are a potential field of pharmacological investigation [94]. The mechanism by which PEA interacts indirectly with CB2R is related to the mechanism of inhibition of FAAH and NAAA. Inhibition of their activity increases the bioavailability of PEA, allowing its sustained interaction with CB2R [31]. In our study, this effect was found to be most favoured by the presence of *Equisetum A.L.* at 50 μg/mL (also in individual form) whose antioxidant activity can reduce oxidative stress in nerve cells. Still, in addition to this, it gave significant results in reducing the effects of FAAH levels and the activity and presence of NAAA. These data align perfectly with the literature on the modulation effects of both FAAH and NAAA on the endocannabinoid system (AEA and 2-AG). As indicated in the literature, FAAH is an enzyme that regulates pain and inflammation [95]. The inhibition of FAAH increases the concentration of endogenous cannabinoids, producing antinociception in several in vitro and animal pain models [96]. According to published research, FAAH is the primary catabolic enzyme that disrupts AEA, converting it into ethanolamine and free arachidonic acid. FAAH is a hydrolase commonly present in the brain, showing a subcellular distribution. Regarding the degradation of PEA, it is dissociated into palmitic acid and ethanolamine through enzymatic action via hydrolysis. Among enzymes, the primary degradation action is carried out by the enzyme NAAA, compared to FAAH [97]. Therefore, *Equisetum A.L.* at 50 μg/mL have shown a modulatory action, inhibiting FAAH and NAAA mechanisms, providing protection and amplifying the analgesic impact identified by PEA at the neurological level. PEA80mesh at 0.2 μM + *Equisetum A.L.* at 50 μg/mL observed reduction values are much lower than individual PEA and H_2_O_2_-induced damage, indicating an excellent analgesic effect. The potential analgesic role of *Equisetum A.L.* at 50 μg/mL already in single form is to be hypothesised thanks to its bioactive components discussed also in the literature in common with other plants. As defined in some studies natural FAAH inhibitors may include botanical extracts, and several natural compounds have been identified as FAAH inhibitors [98,99]. Natural FAAH inhibitors include the following: kaempferol and galangin, two flavonoids; cyanidino-3-glucoside, an anthocyanin belonging to the polyphenol class; biocanine-A, an isoflavone; genistein, also an isoflavone; and daidzein, closely related to genistein [49,100,101]. Kaempferol (3,5,7-trihydroxy-2-(4-hydroxyphenyl)-4H-chromen-4-one) is a flavonoid identified in the genus *Equisetum* as *Equisetum silvaticum L.* [102] and *Equisetum A.L.* [48]. Treatment with kaempferol versus synthetic FAAH URB597 inhibitor has had analgesic and anxiolytic effects in a dose dependent manner [49]. At the same time, also Quercetin documented in the literature in *Equisetum A.L.* [84], is reported to be a weak FAAH inhibitor and it has shown an effective anti-nociceptive effect acting not only on FAAH but also on COX-2 and 5- and 15-lipoxygenases [51,103]. These enzymes can degrade AEA and 2-AG and reduce their presence [104]. This evidence suggests the analgesic potential of the plant used in combination with PEA80mesh, favouring an improvement in its known mechanism of action on FAAH and endocannabinoids. Indeed, studies have shown that PEA can inhibit the action of the FAAH, which has a greater affinity for endocannabinoids such as AEA. Our research has enabled us to confirm that through this action, PEA promotes the availability of AEA, increasing its anti-inflammatory and analgesic effects [105]. In particular, PEA is a false substrate of FAAH; indeed, in the presence of PEA, the enzyme interacts more with this molecule than with endocannabinoids, preserving them and acting as an indirect cannabimimetic compound [106]. The levels of AEA and 2-AG were most strongly increased in the presence of *Equisetum A.L.* at 50 μg/mL both singly and especially in combination with PEA80mesh.

Involving nociceptor-specific ion channels (e.g., TRPV1) and nuclear transcription factors, mechanistic studies suggest PEA may relieve pain (e.g., TRPV1) and nuclear transcription factors (e.g., PPARα in its pharmacodynamic profile) [107]. Its anti-nociceptive and anti-inflammatory actions are terminated by enzyme-mediated hydrolysis, catalysed by the intracellular enzymes FAAH and NAAA [108]. PEA’s analgesic role is attributed to its binding to proteins like PPARα and other intracellular phosphorylation and dephosphorylation mechanisms [107]. A direct receptor-mediated mechanism is necessary for its anti-nociceptive effect at the CNS level. Studies show PEA can activate at least two different receptors, including PPARα. The functional interaction between FAAH and NAAA is still partially understood, but studies suggest a combined effect through concomitant CB2-PPAR-α-TRPV1 activation [109]. From the data obtained in our experiments, as was to be expected, PPARα was found to be significantly higher following treatment with PEA80mesh at 0.2 μM + *Equisetum A.L.* at 50 μg/mL than the forms of PEA examined (*p* < 0.05). Consistent with previous data on FAAH and NAAA, *Equisetum A.L.* at 50 μg/mL recorded similar values to PEA80mesh at 0.2 μM + *Equisetum A.L.* at 50 μg/mL, demonstrating an amplifying role toward the effects promoted by PEA80mesh at 0.2 μM alone. At the same time the lower or higher expression of the pTRPV1 protein was also analysed because the expression of this protein has been found to depend on changes in its phosphorylation state induced by regulatory proteins, including pPKA [31,110]. It was found that its phosphorylation appears to be a requirement for pTRPV1 expression/sensitisation, contributing to pain transmission, inflammation, and neurotoxicity [111]. Therefore, according to these premises, the decreased or increased expression of pPKA and pTRPV1 following treatment with the sample agents was investigated. Consistent with previously obtained data, all samples contributed to reduced expression of pPKA and pTRPV1 concerning H_2_O_2_-induced damage. In particular, PEA80mesh at 0.2 μM + *Equisetum A.L.* at 50 μg/mL recorded the best effects regarding lower activation of this phosphorylation pathway of TRPV1 (*p* < 0.05), contributing to an analgesic response. Again, the amplifying and protective role promoted by *Equisetum A.L.* at 50 μg/mL alone against PEA80mesh at 0.2 μM was confirmed. As discussed above, the beneficial results obtained from treatment with *Equisetum A.L.* alone are potentially due to the bioactive components discussed above. In vivo studies have shown that kaempferol is also able to modulate TRPV1, thus activating the anti-nociceptive mechanism [112] and allowing analgesia to be significantly compared to the control group treated with dimethylsulfoxide (DMSO) [113]. In addition, in another context, *Equisetum A.L.* in combination with other botanical extracts has helped to improve the symptomatology and nociception on an animal model with hyperactive bladder, acting also on TRPV1 [114].

This study presents a multimodal approach to modulating pain transmission by combining PEA80mesh and *Equisetum A.L*. It targets multiple levels of pain transmission by modifying endocannabinoids degradation, activating anti-nociceptive signalling receptors, and reducing TRPV1 and PKA phosphorylation. The combination’s uniqueness is derived from its use of PEA in 80mesh formulation, ensuring better dispersion and bioavailability, and *Equisetum A.L.* with antioxidant and anti-inflammatory activity. When compared to currently available pharmacological treatments for neuropathic pain, such as gabapentin and pregabalin, both of which primarily act by inhibiting voltage-gated calcium channels in the CNS [115], the combination of PEA and *Equisetum A.L*. exhibits a broader, non-neuronal action, modulating glial activation, oxidative stress, and endocannabinoid signalling. While gabapentin is effective in reducing pain in many patients, it is often associated with adverse effects, such as dizziness, fatigue, and cognitive impairment [116]. In contrast, the combination of PEA80mesh and *Equisetum A.L*. demonstrated in vitro tolerability, suggesting a potentially safer profile, especially in chronic settings. Moreover, while gabapentin targets the symptoms [117], the current formulation may also act upstream by preserving BBB integrity and attenuating the cellular mechanisms that initiate and sustain neuroinflammation. Several recent studies have reported the efficacy of PEA, alone or in combination with other bioactive agents, in modulating neuropathic pain mechanisms both in preclinical and clinical contexts [4,118,119,120]. However, to the best of our knowledge, no published study has yet explored the combined effect of PEA80mesh and *Equisetum A.L.* on a co-culture model mimicking BBB–CNS interaction. This adds novelty and translational interest to the present study.

Despite these promising findings, this study has some limitations. Firstly, all results were obtained from in vitro models, which cannot fully replicate the complexity of the in vivo environment. Secondly, while statistical comparisons between the combination and single treatments were performed, formal synergy testing (e.g., isobologram or combination index analysis) and comparisons with reference drugs were not conducted. To address these limitations and assess the translational potential of the formulation, a before–after clinical study has been conducted on patients with chronic pain, evaluating the effects of the same formulation on the quality of life, pain perception, and inflammatory status. This data will be discussed in a future publication and will serve as a bridge between the current in vitro findings and clinical applicability.

## 5. Conclusions

The present study demonstrated that PEA80mesh at 0.2 μM is able to cross the in vitro BBB model, with increased permeability when combined with *Equisetum A.L*. at 50 μg/mL. The combination was well tolerated at both the BBB and CNS cell levels, preserving cell viability and homeostasis, and contributing to the maintenance of BBB integrity under standardised assay conditions. Although these findings are promising, they are limited to an in vitro model and should be interpreted accordingly and integrated with in vivo and clinical studies. The combination modulated key targets involved in nociceptive transmission, such as FAAH, NAAA, CB2R, PPAR-α, and TRPV1, suggesting a potential mechanistic role in the modulation of neuroinflammatory pathways. Notably, *Equisetum A.L*. appeared to enhance the effects of PEA80mesh by supporting endocannabinoid signalling and possibly limiting enzymatic degradation. Together these results provide a foundation for further investigation into the combined use of PEA and *Equisetum A.L*. as a potential strategy for nociceptive modulation.

## Data Availability

The Laboratory of Physiology (F. Uberti) collects raw data and takes appropriate procedures to preserve them forever in a secure system. The corresponding author can provide this study’s data upon reasonable request.

## Abbreviations

The following abbreviations are used in this manuscript:

2-AG	2-arachidonoylglycerol
Adv DMEM	Advanced Dulbecco’s Modified Eagle Medium
Adv DMEM F12	Advanced Dulbecco’s Modified Eagle Medium F12
AEA	Arachidonoylethanolamide or Anandamide
ATCC	American Type Culture Collection
BBB	Blood–Brain Barrier
CB1R	Cannabinoid Receptors 1
CB2R	Cannabinoid Receptors 2
CNS	Central Nervous System
DMEM	Dulbecco’s Modified Eagle’s Medium
EGM-2	Endothelial Growth Medium-2
*Equisetum A.L.*	*Equisetum arvense L.*
FAAH	Fatty Acid Amide Hydrolase
FBS	Foetal Bovine Serum
HBVPs	Primary Human Cerebral Vascular Pericytes
HUVEC	Human Umbilical Vein Endothelial Cells
IL-1β	Interleukin 1β
MTT	3-(4,5-Dimethylthiazol-2-yl)-2,5-diphenyltetrazolium bromide
NAAA	*N*-acylethanolamine-hydrolyzing acid amidase
P/S	Penicillin–Streptomycin
PEA	palmitoylethanolamide
PEA-um	PEA Ultramicronized
PKA	Protein Kinase A
PKC	Protein Kinase C
PNS	Peripheral Nervous Systems
PPARα	Peroxisome Proliferator-Activated Receptor Alpha
pPKA	Phospho-Protein Kinase A
pTRPV1	Phospho-Transient Receptor Potential Vanilloid 1
ROS	Reactive Oxygen Species
RPMI	Roswell Park Memorial Institute medium
TJ	Tight Junction
TNFα	Tumour Necrosis Factor α
TRPV1	Transient Receptor Potential Vanilloid 1
